# Natural selection of immune and metabolic genes associated with health in two lowland Bolivian populations

**DOI:** 10.1073/pnas.2207544120

**Published:** 2022-12-27

**Authors:** Amanda J. Lea, Angela Garcia, Jesusa Arevalo, Julien F. Ayroles, Kenneth Buetow, Steve W. Cole, Daniel Eid Rodriguez, Maguin Gutierrez, Heather M. Highland, Paul L. Hooper, Anne Justice, Thomas Kraft, Kari E. North, Jonathan Stieglitz, Hillard Kaplan, Benjamin C. Trumble, Michael D. Gurven

**Affiliations:** ^a^Department of Biological Sciences, Vanderbilt University, Nashville, TN 37235; ^b^Center for Evolution and Medicine, Arizona State University, Tempe, AZ 85287; ^c^Department of Medicine, University of California, Los Angeles, CA 90095; ^d^Department of Ecology and Evolution, Princeton University, Princeton, NJ 08544; ^e^Lewis Sigler Institute for Integrative Genomics, Princeton University, Princeton, NJ 08544; ^f^School of Life Sciences, Arizona State University, Tempe, AZ 85287; ^g^Department of Psychiatry and Biobehavioral Sciences, University of California, Los Angeles, CA 90095; ^h^Department of Medicine, University of California, Los Angeles, CA 90095; ^i^Universidad de San Simon, Cochabamba, Bolivia; ^j^Gran Consejo Tsimane, San Borja, Bolivia; ^k^Department of Epidemiology, University of North Carolina at Chapel Hill, Chapel Hill, NC 27516; ^l^Economic Science Institute, Chapman University, Orange, CA 92866; ^m^Geisinger, Danville, PA 17821; ^n^Department of Anthropology, University of Utah, Salt Lake City, UT 84112; ^o^Institute for Advanced Study in Toulouse, Toulouse, 31080 France; ^p^Institute for Economics and Society, Chapman University, Orange, CA 92866; ^q^School of Human Evolution and Social Change, Arizona State University, Tempe, AZ 85287; ^r^Department of Anthropology, University of California, Santa Barbara, CA 93106

**Keywords:** natural selection, evolution, Tsimane, genotype–phenotype, health

## Abstract

A growing body of work has addressed human adaptations to diverse environments using genomic data, but few studies have connected putatively selected alleles to phenotypes, much less among underrepresented populations such as Amerindians. Studies of natural selection and genotype–phenotype relationships in underrepresented populations hold potential to uncover previously undescribed loci underlying evolutionarily and biomedically relevant traits. Here, we worked with the Tsimane and the Moseten, two Amerindian populations inhabiting the Bolivian lowlands. We focused most intensively on the Tsimane, because long-term anthropological work with this group has shown that they have a high burden of both macro and microparasites, as well as minimal cardiometabolic disease or dementia. We therefore generated genome-wide genotype data for Tsimane individuals to study natural selection, and paired this with blood mRNA-seq as well as cardiometabolic and immune biomarker data generated from a larger sample that included both populations. In the Tsimane, we identified 21 regions that are candidates for selective sweeps, as well as 5 immune traits that show evidence for polygenic selection (e.g., C-reactive protein levels and the response to coronaviruses). Genes overlapping candidate regions were strongly enriched for known involvement in immune-related traits, such as abundance of lymphocytes and eosinophils. Importantly, we were also able to draw on extensive phenotype information for the Tsimane and Moseten and link five regions (containing *PSD4*, *MUC21* and *MUC22*, *TOX2*, *ANXA6*, and *ABCA1*) with biomarkers of immune and metabolic function. Together, our work highlights the utility of pairing evolutionary analyses with anthropological and biomedical data to gain insight into the genetic basis of health-related traits.

## Significance

Humans have adapted to diverse environments, but few studies have linked regions of the genome with evidence of selection to phenotypes. We generated an integrative dataset of genomic, transcriptomic, and biomarker data for the Tsimane and the Moseten—two indigenous Amerindian populations in Bolivia. From analyses focused on Tsimane individuals, we found evidence for adaptation at genes and traits involved in immunity, which makes sense given that infection has strong effects on physiology and fitness in this population. Using phenotypic data from both populations, we were able to link genotypes to the immune and metabolic traits that are potentially advantageous. This study expands our knowledge of natural selection in Amerindians and uncovers previously undescribed loci of evolutionary and biomedical relevance.

Since migrating out of Africa, humans have inhabited most terrestrial regions of this planet and have been exposed to diverse diets, climates, pathogens, and other environmental challenges. Over time, these ecological selection pressures have presumably driven many instances of local adaptation, yet scientists have so far only uncovered a handful of recent genetic adaptations with validated phenotypic consequences. Examples include mutations in the lactase enzyme gene, which allow adults of select European, African, and Middle Eastern ancestries to digest milk (1–3); mutations in the red blood cell production gene *EPAS1*, which enable Tibetan highlanders to persist at high altitudes (4, 5); and mutations in fatty acid desaturase genes, which allow Greenlandic Inuit to subsist on a meat and fish-focused diet rich in omega-3 fatty acids (6). The relative paucity in the literature of positively selected loci with clear phenotypic connections is largely due to difficulties in sampling human populations of interest, robustly detecting signals of selection (7), and following up on loci identified by statistical analyses with functional or physiological assays. Overcoming these challenges is important for understanding how genetic variation shapes fitness-related traits in our species, which is key for both understanding human evolution and for improving human health.

The present study seeks to understand the genomic and phenotypic consequences of natural selection in the Tsimane—an indigenous Amerindian population inhabiting lowland Bolivia. We focus on the Tsimane because: 1) they inhabit a high pathogen environment, which is known to affect physiology, fertility, and mortality (8–11), leading to clear predictions about the types of selection pressures that were likely important in recent history; 2) they have partnered with an integrated anthropological and biomedical study for nearly two decades (12), which has resulted in a wealth of phenotypic data that could be linked to genetic variation; and 3) more broadly, indigenous peoples of the Americas are one of the most underrepresented groups in genetic research (13, 14). Only a handful of previous genomic studies have explored positive selection in population-scale samples of contemporary Native Americans (e.g., refs. 15–18), and even fewer have linked selection signals with phenotypes (19–21). A notable exception is recent work from Asgari and colleagues (19), who identified a previously uncharacterized missense variant in *FBN1* in Peruvians that is under positive selection and is associated with microfibril structure and a ~2-cm reduction in height. This work highlights the potential for genetic studies in underrepresented populations to uncover phenotypically relevant loci. The value of such analyses is especially salient in light of important epidemiological observations made about the Tsimane over the past decade: exceptionally low rates of cardiovascular and cognitive diseases, benign prostate hyperplasia, and COVID-19 mortality (12, 22, 23, 76).

The Tsimane largely maintain a traditional, subsistence-level lifestyle supported through slash‐and‐burn horticulture, fishing, hunting, and seasonal foraging (12). They have inhabited the Bolivian Piedmont area for at least several hundred years, but the peopling of this region is poorly understood, as is the genetic history of the Tsimane themselves (24, 25). Because they reside in rural villages located within the warm and humid neotropics, the Tsimane experience a high pathogen burden relative to populations inhabiting more northern latitudes and/or urban environments. More than two-thirds of adults suffer from intestinal helminths at any given time, and about half of all adults present with respiratory infection (10, 26). Not surprisingly, infections are the primary source of morbidity and mortality (11).

This high pathogen burden shapes many immune and other physiological traits in the Tsimane. Relative to individuals living in the Global North, the Tsimane exhibit elevated levels of C-reactive protein (CRP), various immunoglobulins, B cells, natural killer cells, and eosinophils (26, 27). Previous work has also shown that persistent infection impacts energetics and fertility (e.g., resting metabolic rate, female age at first birth, and female interbirth intervals) (8–10), and may contribute to the extremely low rates of cardiometabolic disease observed in the population (12, 22, 28, 29). However, because only a handful of genetic studies have been conducted in the Tsimane—all of which have focused on candidate genes, used small sample sizes, and/or sampled areas where Tsimane coreside with other ethnolinguistic groups (24, 25, 30, 31)—it is unclear whether and to what degree the rare physiological traits exhibited by the Tsimane are genetically based products of natural selection. Answering this question is relevant for understanding the evolution and genetic basis of health in a unique Native American group, as well as human evolutionary processes more broadly.

Here, we use a three-pronged approach to test the hypothesis that traits that shape, or are shaped by, immune defenses are under selection in the Tsimane. First, we used genome-wide genotype data to scan 203 Tsimane genomes for evidence of selective sweeps. Second, to functionally validate the candidate regions identified by our selection scans, we drew on existing biomarker and blood mRNA-sequencing (mRNA-seq) data to ask if any of these regions contained loci associated with 1) gene expression levels in whole blood (n = 242) and/or 2) relevant physiological biomarkers (average n = 1,214). To maximize power, these follow-up analyses included data from a nearby, genetically and ethnolinguistically related population, the Moseten (12, 24) (Moseten individuals comprise 36% and 14% of the mRNA-seq and biomarker datasets, respectively). We note that we did not have a large enough set of Moseten genotypes to conduct separate selection scans for this population, and thus this population is only included in phenotypic follow-up analyses (similar to the approach of ref. 6). Finally, given recent evidence that many adaptive traits are shaped by simultaneous selection on numerous genetic variants (32, 33), we also tested for so-called “polygenic adaptation” on 22 quantitative immune traits (34), and on immune responses to 16 viruses (35).

In total, our analyses identified 21 nonoverlapping regions that are candidates for selective sweeps, as well as several immune traits that show evidence for polygenic selection (e.g., CRP levels and the response to coronaviruses). Our candidate regions included or were near genes that code for key immune [e.g., *PSD4*, *MUC21*, *MUC22*, and *TOX2* (36–39)] and metabolic molecules, including several major regulators of lipids that contribute to cardiometabolic disease [e.g., *ANXA6* and *ABCA1* (40–45)]. Importantly, we were able to explore the functional consequences of selected loci by linking the regions mentioned above (that contain *PSD4*, *MUC21* and *MUC22, TOX2, ANXA6*, and *ABCA1*) to gene expression and/or biomarker variation. Together, our work leverages an integrative dataset from a genetically understudied Native American group to identify genotype–phenotype connections of evolutionary and biomedical significance.

## Results

### Population Genetics of the Tsimane.

**Fig. 1.**
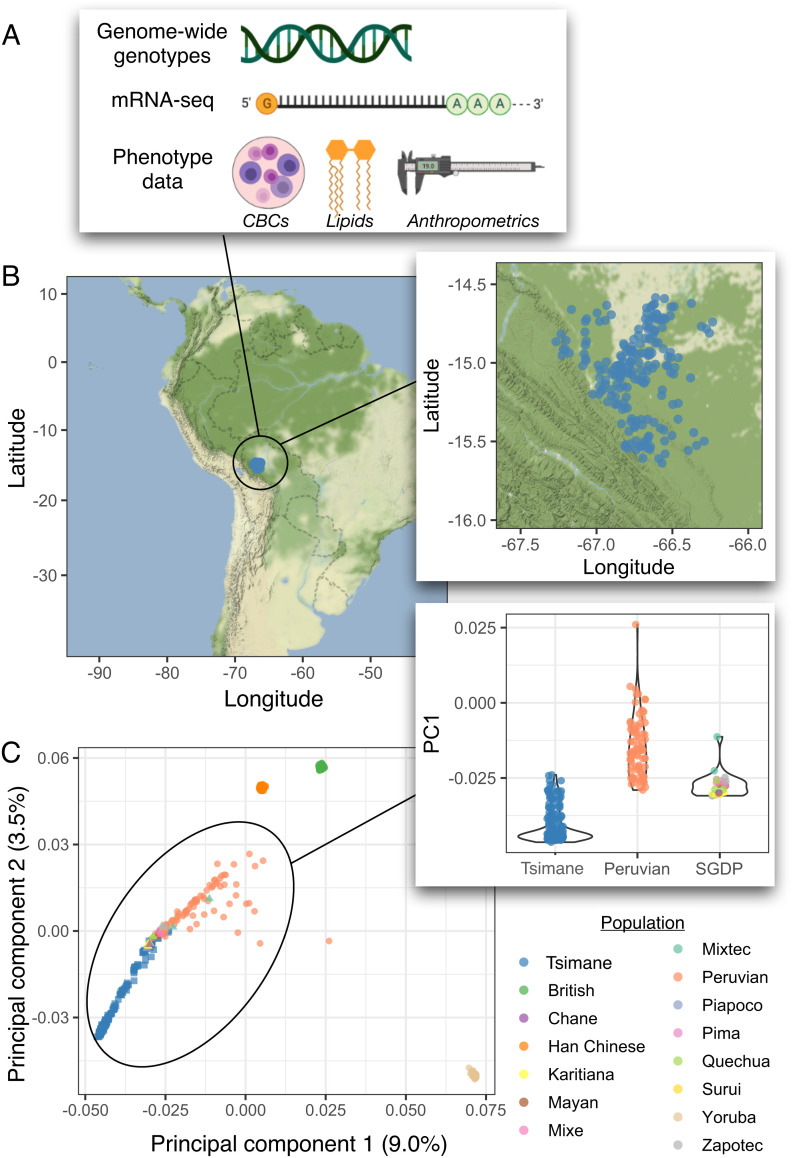
Study overview. *A*) Overview of data types: MEGA genome-wide genotypes, mRNA-seq, and phenotype data of several types (e.g., anthropometrics, lipid panels, complete blood counts; see *SI Appendix*, Table S5). *B*) Specific sampling locations within Bolivia for unrelated Tsimane samples (n = 203). *C*) Results from a PCA including Tsimane samples as well as 1) Han Chinese, Peruvians, Yoruba, and British individuals from 1000 Genomes and 2) all Central and South American individuals from the SGDP. Samples are colored by their population of origin and shapes denote which study generated the data (circles = 1000 Genomes, triangles = SGDP, squares = this study). *Inset* shows values for principal component 1 (PC 1) stratified by population and/or study for visualization. A version of this figure that includes Moseten samples is presented as *SI Appendix*, Fig. S7.

We genotyped 1,080 Tsimane individuals using the Infinium Multi-Ethnic Global Array (MEGA) (Fig. 1). Before proceeding to selection analyses, we explored the genetic distance between the Tsimane and other South American groups and the degree of recent admixture within the Tsimane, as admixture can complicate selection inference (46). To do so, we first applied principal component analyses (PCA). We pruned our dataset to include only unrelated individuals genotyped at 1,754,170 high-quality single nucleotide polymorphisms (SNPs) (coefficient of relatedness *r *< 0.125, n = 203; see *Methods*). We merged these data with publicly available whole-genome sequences from the 1000 Genomes Project (47) and the Simons Genome Diversity Project (SGDP; focusing on populations from Central and South America) (48). As expected, Tsimane clustered most closely with unadmixed individuals from South American populations sampled by the SGDP (e.g., Quechua, Karitiana, and Piapoco; Fig. 1). No Tsimane individuals clustered with Peruvian individuals sampled by the 1000 Genomes Project that contain high amounts of European admixture (Fig. 1).

**Fig. 2.**
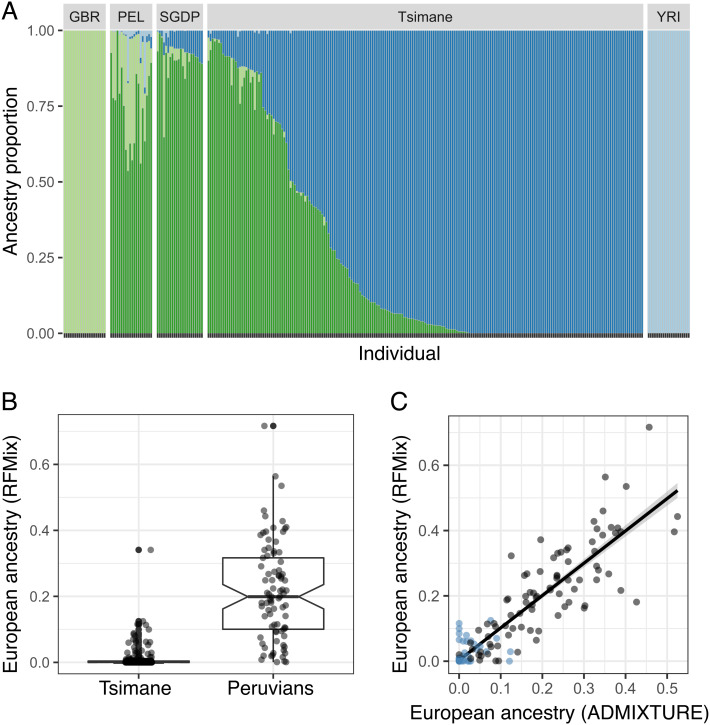
Limited evidence for European admixture within the Tsimane. *A*) Admixture estimates from the program ADMIXTURE for Tsimane individuals, as well as 1) all Central and South American individuals from the SGDP and 2) Peruvian (PEL), British (GBR), and Yoruba (YRI) individuals from the 1000 Genomes Project. All 1000 Genomes populations were randomly down sampled to 20 individuals for plotting. Results are shown for K = 4; results for other values of K are presented in *SI Appendix*, Fig. S1. *B*) Admixture estimates from the program RFMix for Tsimane as well as Peruvian individuals as a control, since admixture for these individuals has been characterized elsewhere. *C*) Comparison of estimated European admixture proportions from the programs RFMix versus ADMIXTURE for Tsimane (blue) and Peruvian (black) individuals (R^2^ = 0.84, *P* < 10^−16^). Both approaches suggest minimal European admixture in the Tsimane.

To more directly test for European as well as West African admixture, both of which are common in South American populations as a result of colonization and the transatlantic slave trade (15, 49, 50), we used two additional statistical approaches. First, we performed nonhierarchical clustering on our dataset of unrelated Tsimane individuals using ADMIXTURE (51) (exploring values of K = 3 to 6). Second, we performed local ancestry assignment using RFMix to assign each chromosomal segment for each sample to its most likely ancestral source population (52). Global ancestry proportion results from RFMix are consistent with those from ADMIXTURE (R^2^ = 0.84, *P* < 10^−16^; Fig. 2 and *SI Appendix*, Fig. S1). Consistent with our PCA results, both approaches suggest minimal evidence of European ancestry (mean ± SD of RFMix estimate: 4.276 × 10^−3^ ± 0.014) or African ancestry (mean ± SD: 7.453 × 10^−5^ ± 7.819 × 10^−4^; Fig. 2).

### Genome-Wide Scans for Positive Selection.

**Fig. 3.**
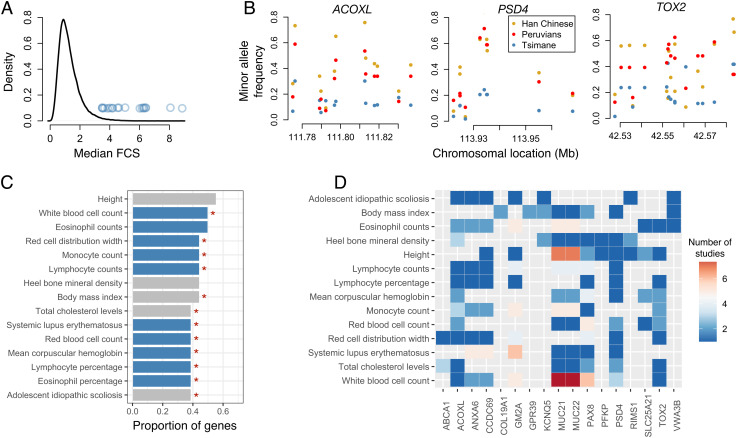
Genes under selection in the Tsimane. *A*) The genome-wide distribution of evidence for positive selection (summarized as the median FCS per 50-kb window). Blue dots represent 21 nonoverlapping candidate regions that passed our significance criteria. *B*) Examples of allele frequency variation within select candidate regions. Minor allele frequencies are plotted for the Tsimane relative to two reference populations from the 1000 Genomes Project. The name of the gene overlapping a given candidate 50-kb window is listed. *C*) The proportion of overlapping candidate genes associated with a given phenotypic trait through GWAS (for traits associated with more than six genes). Blue bars indicate immune-related traits, and red stars indicate traits for which there are more associated genes in our overlapping candidate list than expected by chance. *D*) For traits associated with more than six genes, the heatmap shows the number ofstudies that have associated each candidate gene with a given trait.

To ensure that small amounts of European admixture did not bias our analyses, we excluded seven individuals with >5% European ancestry inferred from RFMix, and we masked SNPs in any remaining individuals’ genomes that were putatively inherited from a European ancestor (see *Methods*). To identify signals of strong selective sweeps, we searched for variants with both high allele frequencies and extended haplotype homozygosity in the Tsimane relative to reference populations from the 1000 Genomes Project. More specifically, we calculated 1) the integrated haplotype score (iHS) (53), 2) the population branch statistic (PBS; using Han Chinese and unadmixed Peruvians as outgroups, *SI Appendix*, Table S1) (5), and 3) the cross population extended haplotype homozygosity (XP-EHH) score (using Han Chinese as the only outgroup because of the low number of unadmixed Peruvians) (54). For each SNP, we summarized the genome-wide ranks of all three statistics using Fisher’s combined score (FCS) (55); we then identified 50-kb windows with consistently high numbers of outliers (see *Methods* and *SI Appendix*, Fig. S2). This procedure left us with 39 50-kb regions, which we further collapsed into 21 nonoverlapping candidate regions (Fig. 3 and *SI Appendix*, Tables S2 and S3).

In addition to positive selection, allele frequency and homozygosity patterns in a population are determined by neutral and demographic processes. To confirm that our results could not be explained by these forces alone, we used the demographic models and the general approach of (16) to simulate genomic data in the absence of selection (using fastsimcoal2 (56), see *Methods* and *SI Appendix*, Table S4 for demographic model parameters). Across 100 simulated genomic datasets, we rarely observed selection statistics as extreme as those calculated from the real data. More specifically, when we calculated the median FCS for each of our 21 candidate regions, we found that these values were more extreme than 99.5% ± 0.76% of the combined FCS distribution from 100 simulated datasets (*SI Appendix*, Fig. S3).

Having confirmed that our 21 candidate regions were probably not evolving neutrally, we next sought to understand their genic content and biological function. These regions overlapped 18 unique genes (*SI Appendix*, Table S5), including immune-related genes. For example, *MUC21* and *MUC22* are mucin family genes involved in protecting the epithelium from microorganisms (57), while *PSD4* plays a key role in major histocompatibility complex (MHC) class II antigen presentation (58). Our candidate gene list also included major players in lipid metabolism such as *ANXA6*, which modulates triglyceride accumulation and storage (44, 45), and *ABCA1*, which binds and transports cholesterol and is thus a key determinant of total and high-density lipoprotein (HDL) cholesterol levels (58).

To further understand the putative biological functions of genes overlapping our candidate regions, we used public catalogs of genome-wide association study (GWAS) summary statistics to ask whether these genes had been previously associated with phenotypic traits. As expected, given the Tsimane’s high pathogen environment, we found that many of our candidate genes are associated with immune-related traits such as eosinophil counts (9/18 genes) and percentages (7/18), lymphocyte counts (7/18), and overall white blood cell counts (9/18; Fig. 3). For phenotypic traits that were associated with six or more candidate genes, we tested whether the proportion of associated genes represents a significant enrichment over chance expectations, and found that this was indeed true for most traits (Fig. 3 and *SI Appendix*, Table S6).

### Molecular and Phenotypic Effects of Candidate Genes.

To understand the phenotypic effects of genetic variation within our candidate regions, we drew on whole-blood mRNA-seq data (n = 242) and immune and metabolic biomarker data for Tsimane and Moseten individuals. Specifically, our biomarker data included: lipid panels measuring low- and high-density lipoproteins, total cholesterol, and triglyceride concentrations (22, 28); fasting blood glucose (22); five-part white blood cell differential measuring total and proportional basophil, eosinophil, lymphocyte, neutrophil, and monocyte counts (59); anthropometric measures of body fat percentage, body mass index, and waist circumference; and diastolic and systolic blood pressure (60, 61) (average n = 1,214; see *SI Appendix*, Table S7 for biomarker-specific sample sizes). For the 2,329 SNPs that fell within our candidate regions and passed our filtering criteria (see *Methods*), we tested for correlations between genotype and each biomarker as well as between genotype and gene expression levels (also known as “expression quantitative trait loci” or eQTL). While our gene expression and biomarker datasets include both Tsimane and Moseten individuals to boost power, our results are very similar whether Moseten individuals were included or excluded (*SI Appendix*, Figs. S4 and S5 and Tables S8 and S9). We focus here on results that were statistically significant in both analyses.

**Fig. 4.**
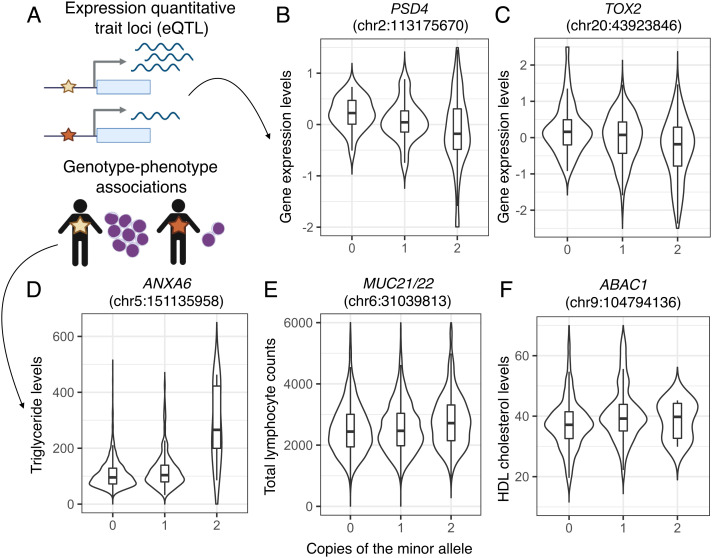
Phenotypic effects of genetic variation under selection in the Tsimane. *A*) Analysis overview. For each candidate region, we performed association mapping to test whether SNPs in the region were associated with 1) gene expression (for cases where the candidate gene was expressed in whole blood; *SI Appendix*, Fig. S6) or 2) 21 metabolic and immune traits (*SI Appendix*, Table S5). In all cases, gene expression and phenotypes were modeled linearly and assume an additive model. *B* and *C*) Results for candidate regions with a significant eQTL. *D*–*F*) Results for candidate regions with a significant genotype–phenotype association. The x-axis shows copies of the minor allele, and the y-axis shows normalized gene expression levels or phenotypic measures (units for triglyceride and HDL cholesterol levels = mg/dL). The number of individuals in each genotypic class (0, 1, or 2) are as follows: *PSD4* (132, 90, 20), *TOX2* (77, 121, 44), *ANXA6* (2,514, 329, 12), *MUC21/22* (2,536, 2,618, 842), and *ABCA1* (2,381, 298, 8). Detailed results are provided in *SI Appendix*, Tables S6–S8.

Nine of the 18 candidate genes are expressed in whole blood and could thus be tested for eQTL (*SI Appendix*, Fig. S6). As expected, most of the genes expressed in blood have some reported immune function; one notable exception was *PFKP*, which codes for the platelet isoform of the enzyme involved in the rate-limiting step of glycolysis (62). Through our eQTL analyses, we found evidence that genetic variation under selection regulates the expression of *TOX2* [SNP = chr20:43923846 (hg38), beta = −0.352, *P* = 8.57 × 10^−5^] and *PSD4* (SNP = chr2:113175670; beta = −0.204, *P* = 0.044; Fig. 4, *SI Appendix*, Fig. S5 and Table S8). Given that many of the candidate genes are not expressed in whole blood and are presumably active in other tissues, we caution that absence of evidence in this analysis is not evidence of absence in terms of a selected variant regulating gene expression.

Using the biomarker dataset, we also identified regulatory SNPs 1) within *ABCA1* that correlate with HDL cholesterol levels (SNP = chr9:104794136 (hg38); beta = 2.858, *P* = 3.82 × 10^−6^), 2) within *ANXA6* that correlate with triglyceride levels (SNP = chr5:151135958; beta = 22.851, *P* = 2.04 × 10^−7^) and total cholesterol levels (SNP = chr5:151135958; beta = 9.812, *P* = 7.47 × 10^−5^), and 3) near *MUC21 and MUC22* that correlate with total lymphocyte counts (SNP = chr6:31039813; beta = 119.7642, *P* = 7.88 × 10^−5^; Fig. 4 and *SI Appendix*, Fig. S5 and Tables S9 and S10). When analyzing Tsimane samples alone, we also find evidence for an association between regulatory variants in *SLC25A21* and fasting blood glucose levels (SNP = chr14:36702706; beta = 3.306, *P* = 7.77 × 10^−5^). The associations between selected variants in *ABCA1* and HDL cholesterol levels (Fig. 4*F*) and between selected variants in *ANXA6* and cholesterol and triglyceride levels (Fig. 4*D*) are notable given the fundamental role these genes play in lipid regulation and transport.

### Polygenic Selection on Immune-Related Traits.

Recent theoretical and empirical work (32, 33, 63) suggests that most adaptive traits are shaped by simultaneous selection on numerous genetic variants, rather than strong selection at a single locus. While so-called “selective sweeps” at single loci are still undoubtedly important in human evolution (64), we were motivated to also explore the contribution of polygenic adaptation to immune-related traits in the Tsimane. To do so, we used two a priori defined sets of genomic regions that have been associated with immune-related traits: 1) SNPs associated with 22 quantitative immune traits via multiancestry GWAS in the UK Biobank (downloaded from https://pan.ukbb.broadinstitute.org/) and 2) genes whose products are known to physically interact with 16 families of viruses (obtained from ref. 35). For each trait, we compared the distribution of selection statistics in nonoverlapping, 50-kb genomic windows containing trait-associated loci to that of randomly sampled windows matched for SNP density, recombination rate, and background selection (as in ref. 65).

**Fig. 5.**
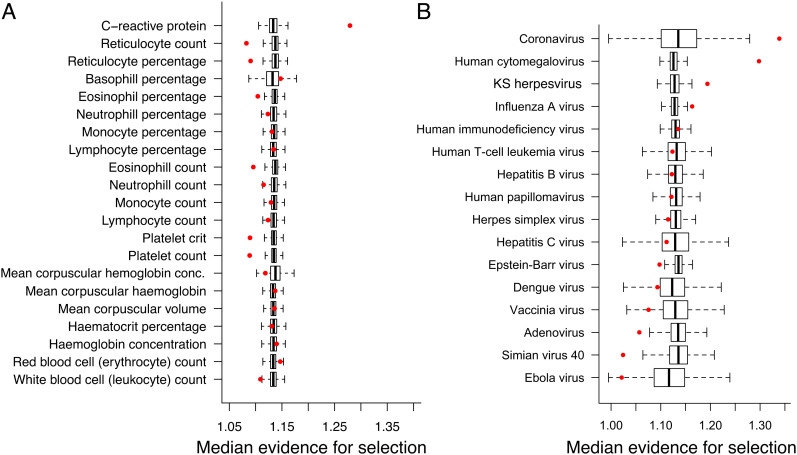
Polygenic selection on immune-related traits in the Tsimane. The y-axis shows the median evidence for selection (FCS) for *A*) regions associated with quantitative immune traits in the UK Biobank or *B*) regions containing genes that are known to interact with particular viral families. Red dots indicate values for the observed data, while boxplots represent the distribution of values from 10,000 randomly drawn datasets.

We observe minimal evidence for selection on quantitative immune traits in our analyses that rely on summary statistics from the UK Biobank, with only CRP levels exhibiting significantly more evidence for selection relative to chance (*SI Appendix*, Table S11 and Fig. 5). Out of 16 tested viral families, proteins that interact with coronaviruses, Kaposi's sarcoma herpesvirus (KSHV), cytomegalovirus (CMV), and influenza A virus show evidence of polygenic selection (*SI Appendix*, Table S12 and Fig. 5). All of these viruses are associated with morbidity and/or mortality and are known to be present in Amerindian groups or in the Tsimane specifically (66–69).

## Discussion

Our study uncovers genomic signatures of natural selection and their phenotypic consequences in an Amerindian population inhabiting an environment with a relatively high pathogen burden. Previous work with the Tsimane has shown that their high pathogen burden is a potent selective force affecting multiple fitness components: more than half of all Tsimane deaths are attributed to infection (11) and intestinal geohelminth infection is associated with female fertility (specifically, roundworm infection predicts higher fertility while hookworm infection predicts lower fertility) (10). Compared with populations with a lower pathogen burden, the Tsimane exhibit elevated levels of many immune biomarkers such as CRP, immunoglobulins, and eosinophils (26, 27). This previous work led us to hypothesize that genes involved in immune defense and investment have been historically under selection in the Tsimane, which we tested with two complementary approaches. First, we scanned Tsimane genomes for signatures of classic selective sweeps, where beneficial mutations at single loci quickly rise to high frequency; we also validated these candidate sweep loci with phenotypic data (Fig. 4). Second, because single-locus selective sweeps may be relatively rare in human evolution (32, 63), we searched for evidence of polygenic selection on immune-related traits. In particular, we used recently proposed methods that test for an enrichment of selection signals at a priori defined sets of trait-associated regions, and that have been successfully applied to diverse populations (65).

We observe patterns indicative of ongoing or partial selective sweeps at 21 candidate regions encompassing 18 genes. This candidate gene list is enriched for involvement in immune-related traits (Fig. 3), such as eosinophil percentages. This enrichment is particularly interesting given that eosinophils are an important cell type for fighting helminth infections, which affect ~2/3 of all Tsimane (9). It was previously believed that the elevated Tsimane eosinophil percentages (~20%) relative to the United States (<5%) (23) were largely a plastic response to high helminth burdens. Using our dataset, we estimate a heritability of 0.193 (SE = 0.046) and 0.162 (SE = 0.049) for eosinophil percentages and total counts, respectively [using GCTA (70), n = 1227]. Combined with our selection analyses, these results suggest there is additive genetic variance for eosinophil production on which selection is acting. Future work is needed to understand the relative contribution of, and interactions between, genetics and pathogen-induced plasticity in shaping Tsimane eosinophil profiles.

Our candidate gene list included many key players in immune defense. For example, *MUC21* and *MUC22* are members of the mucin family, which help protect epithelial tissues from pathogens as well as the host’s own microbiota (39), and *PSD4* is one of a handful of genes in the pathway controlling MHC class II antigen presentation in dendritic cells (36). This particular finding aligns with previous studies of natural selection that have also identified MHC genes as key targets, including in Native American groups (65, 71, 72). We also find evidence for selection at *TOX2*—a transcription factor that is essential for the development of T cells (37) and natural killer cells (38), both of which are elevated in the Tsimane compared with post-industrial populations experiencing lower infectious disease burden (26). Importantly, we were able to link putatively selected variants at *MUC21/22*, *PSD4*, and *TOX2* with variation in blood gene expression levels and/or immune biomarkers (Fig. 4 and *SI Appendix*, Tables S8–S10). This phenotypic validation is essential for understanding the functional consequences of natural selection, for prioritizing loci for follow-up work, and for separating false- and true-positive signals. As complementary phenotypic information is rarely available in genomic studies of natural selection, the current study highlights a major strength of combining genomics with integrative, long-term anthropological research (12).

Somewhat unexpectedly, we find that many genes involved in lipid metabolism are under selection in the Tsimane. For example, *ABCA1* is under selection and is associated with HDL cholesterol levels, such that the major allele at the tag SNP (chr9:104794136) predicts reduced HDL (Fig. 4 and *SI Appendix*, Tables S9 and S10). *ABCA1* is a rate-limiting factor for HDL assembly (40) and loss of function of *ABCA1* in mouse models results in almost complete absence of HDL (41). In humans, *ABCA1* has been linked with atherosclerosis and ischemic heart disease (42, 43), which is notable given that the Tsimane exhibit the lowest levels of coronary artery calcification reported for any population (22). That being said, Tsimane show very low HDL levels: 57% of adults would be considered at-risk given the usual recommendations of >40 mg/dL (22, 73).

We also found evidence for selection on *ANXA6*, which controls cholesterol transport (44). Knockdown and overexpression experiments in adipocytes have shown that *ANXA6* directly modulates triglyceride levels (45), but to our knowledge *ANXA6* has not been previously linked to triglyceride levels in humans. Here, we find a significant and sizeable association: individuals with two copies of the minor allele at the *ANXA6* tag SNP (chr5:151135958) exhibit triglyceride levels that are 2.7 times higher than individuals with two copies of the major allele (Fig. 4 and *SI Appendix*, Tables S9 and S10). While this tag SNP does have an appreciable worldwide minor allele frequency (41%), we note that it could be linked to population-specific genetic variation in the Tsimane. More generally, we cannot claim that any of the SNPs highlighted in the present study have causal effects on phenotypes in the Tsimane. We also note that the genotype–phenotype associations we uncover are on average small in terms of their effect sizes, but this is not unexpected given that 1) variants with large, detrimental effects are selected against and are thus expected to be rare and 2) complex immune and metabolic traits are both highly polygenic and strongly influenced by nongenetic factors (e.g., diet, infection status, etc.) Future studies using whole-genome sequencing as well as gene editing techniques and/or functional assays are needed to identify causal loci and to understand their effects on biological processes in the absence of environmental heterogeneity.

Our polygenic selection analyses revealed significant selection on genes involved in the response to coronaviruses, CMV, KSHV , and influenza A (Fig. 5 and *SI Appendix*, Table S11). These are all viruses that are known to be at especially high prevalence, or to have disproportionate effects, in Native Americans. CMV prevalence is generally high in South America (68), and KSHV prevalence is extremely high (~75%) in Amazonian Amerindians (69). Further, US Native Americans are at roughly two-fold higher risk of serious flu complications, and are the only macroethnic group considered to be uniquely at risk (74). Of current public health interest, COVID-19 spread widely among the focal Tsimane population (75), with a cumulative incidence of ~75%. Yet based on our follow-up analyses, case fatality rates are much lower than those reported in the United States and China (76). Ongoing work is addressing why severe outcomes appear to be less frequent, with possible explanations being that previous exposures to coronaviruses have shaped the Tsimane genome and immune system in ways that might promote cross-reactivity to COVID-19, or that helminth infection may mitigate severe outcomes (77–79).

Our polygenic analyses also uncovered selection on loci related to CRP levels (Fig. 5 and *SI Appendix*, Table S12), which are elevated in the Tsimane relative to post-industrial populations, especially at earlier ages (26, 27) and in certain individuals (26). It was previously speculated that this inter-individual variation points to a partial genetic basis for CRP levels (30), which we confirmed by estimating a non-zero heritability of 0.095 ± 0.060 [using GCTA (70), n = 823]. We were surprised that no additional quantitative immune traits exhibited significant evidence for polygenic selection, and we speculate that this may reflect the limitations involved in using summary statistics derived from GWAS focused on individuals of European ancestry (UK Biobank). Nevertheless, we believe it is important to complement analyses of selective sweeps with approaches that can address other modes of selection.

In summary, we find evidence for the hypothesis that pathogens have been a strong selective force in the Tsimane, as is the case for many human populations (80) including other Native American populations (65, 71, 72). Our findings lead to several future directions. First, whole-genome sequencing can help evaluate the phenotypic effects of additional variants, including population-specific variants that are not captured on the genotyping arrays we used here. Second, functional genomic datasets can clarify how selected variants affect gene regulation (e.g., by measuring chromatin accessibility, transcription factor binding, or DNA methylation levels). Experimental techniques such as reporter gene assays or CRISPR-Cas9-based editing could also assist in identifying causal variants and understanding their effect sizes in the absence of environmental complexity. Third, a genotype x environment approach can shed light on how current infection status modulates the genotype–phenotype associations documented here. More generally, our work highlights the utility of pairing genomic analyses with a strong foundation of ecological and anthropological knowledge, as well as the insights into health-related traits that arise from studying underrepresented groups and leveraging evolutionary analyses.

## Materials and Methods

### Population Overview.

The Tsimane are one of >35 indigenous groups in Bolivia. They inhabit >90 villages in the Beni and La Paz departments, with a population size estimated at ~17,000 individuals. Tsimane practice slash-and-burn horticulture supplemented with fishing, hunting, and seasonal foraging (12). Most Tsimane have retained this subsistence-level lifestyle with relatively minimal impacts from acculturating forces (12, 81). As a result, we expected that power to identify loci involved in local adaptation would be especially robust in this population, because 1) we expected low levels of European admixture and 2) the population still inhabits a high pathogen environment similar to that in which the selective events we are studying occurred. Extensive descriptions of Tsimane lifestyle, culture, and health have been published elsewhere (see ref. 12 and references therein).

While this study focuses on understanding how natural selection has shaped genetic and phenotypic diversity in the Tsimane, we also included samples from a neighboring indigenous horticulturalist population, the Moseten, in some analyses. The Moseten and Tsimane inhabit adjacent territories, speak very similar languages that are part of an isolate language family (82, 83), and exhibit many cultural similarities. A detailed description of the cultural histories of the Tsimane and Moseten is provided by ref. 81, and several papers have been published by members of the Tsimane Health and Life History Project (THLHP) documenting biomedical and anthropological features of both groups (12, 61, 84). Our population genetic analyses revealed that there is relatively minimal, but detectable, genetic distance between the Tsimane and Moseten (*SI Appendix*, Fig. S7). We therefore did not include Moseten individuals in our evolutionary analyses, and we could not perform a separate evolutionary analysis for this population due to sample size limitations. However, we did include Moseten individuals in eQTL and genotype–biomarker association analyses to boost sample size (Fig. 4 and *SI Appendix*, Tables S8–S10); these analyses all explicitly controlled for any genetic structure between Tsimane and Moseten individuals using statistical methods that are well established for GWAS of structured populations (85) (see *Mapping Genotype–Phenotype Correlations at Candidate Regions*). We also repeated all association analyses with and without Moseten individuals to confirm our results (*SI Appendix*, Tables S8–S10).

Informed consent was obtained from all participants after describing the study in their language of choice (Tsimane or Spanish for Tsimane; Spanish for Moseten). Our work was approved by the institutional review boards at the University of California, Santa Barbara and the University of New Mexico (#3-21-0652 and #07-157, respectively). All work was also discussed with and approved by the Tsimane government (Gran Consejo Tsimane), community leaders, and the study participants themselves. We also developed materials to communicate study results to participant communities, which are posted at https://github.com/AmandaJLea/Tsimane_selection.

### Blood Sample Collection, DNA Genotyping, and Genotype Filtering.

Between 2006 and 2015, 1,286 blood samples were collected from individuals belonging to the Tsimane (n = 1,112) and Moseten (n = 174) communities by the THLHP (12). Venous blood samples were collected in anticoagulant tubes by Bolivian medical professionals and immediately frozen in liquid nitrogen. Upon export to the United States, samples were stored at −80 °C.

DNA extraction and SNP genotyping on the Infinum MEGA were performed by the University of Texas Health Sciences Center at Houston. Genotype clustering was performed with Illumina’s Genome Studio. SNPs that did not meet quality control thresholds were manually inspected and redefined as needed. In total, 1,754,170 SNPs (out of an attempted 1,779,819) were successfully genotyped. To assess reproducibility, 14 samples were processed in duplicate and the mean concordance between technical replicates was estimated at 99.99%.

We used Plink (86) to filter for high-quality SNPs and samples and to perform linkage disequilibrium (LD) filtering. We then used PC-Relate (87) to remove close relatives (kinship coefficient > 0.125, corresponding to third degree relatives), leaving us with a dataset of 203 Tsimane individuals. We harmonized this dataset to the 1000 Genomes Phase 3 call set (47) and filtered for SNPs that could be unambiguously merged between the two datasets using Genotype Harmonizer (88) (n = 693,720 biallelic SNPs). Finally, we phased our filtered SNP calls using Shape-IT v2.r904 (89). Additional details on genotype filtering and processing are provided in the *SI Appendix*, *Materials and Methods*.

### Principal Component and Admixture Analyses.

We merged our filtered, phased data with 1000 Genomes Phase 3 data (47) as well as data from the SGDP (48). We performed further LD filtering and then conducted PCA using Plink (86) (Fig. 1). Next, we used two standard approaches to test for potential European and West African admixture. First, we used the program ADMIXTURE (51) to estimate the proportion of the genome originating from K populations for each individual, with K being specified a priori. We tested K = 3 to 7 and found that K = 3 produced the lowest cross-validation error (these results are presented in Fig. 2, see also *SI Appendix*, Fig. S1). Second, we generated local ancestry assignments using RFMix (52), with possible ancestry assignments of British/European, Yoruba/West African, and Native American. Using both analysis approaches, we observed essentially no evidence for African ancestry within the Tsimane and minimal evidence of European ancestry (Fig. 2 and *SI Appendix*, Fig. S1). For all downstream selection analyses, we pruned our set of 203 unrelated individuals down to 196 individuals with >95% Native American ancestry inferred from RFMix, and we masked SNPs in regions that were not confidently assigned to Native American ancestry. These analyses are further detailed in the *SI Appendix*, *Materials and Methods*.

### Genome-Wide Scans for Positive Selection.

We identified genomic regions under recent positive selection in the Tsimane using an outlier approach and three statistics: the iHS (53), the PBS (5), and the XP-EHH score (54). These approaches can detect selection on the order of thousands to tens of thousands of years ago (64). Specifically, the iHS compares patterns of linkage disequilibrium within a population for haplotypes carrying the derived versus ancestral SNP at a given locus, while the PBS and XP-EHH approaches compare allele frequencies and haplotype structure, respectively, between the Tsimane and one to two reference populations. Using three statistics that each look for distinct signals in the data and rely on different aspects of the data (e.g., iHS and XP-EHH analyze phased haplotypes, while PBS analyzes allele frequencies), we reasoned that we could recover robust signals of selection and avoid false positives induced by the processing steps or assumptions required for any individual test. For PBS, we used Han Chinese and unadmixed Peruvians from 1000 Genomes as comparison populations; for XP-EHH, we used Han Chinese because there were a relatively small number of unadmixed Peruvian individuals.

iHS and XP-EHH values were calculated from the phased, admixture-masked data using the R package REHH (90), after derived/ancestral state was assigned from six-way alignments at each locus (47). Derived/ancestral state was added using the *fill-aa* tool from VCFtools (91). Only variants with minor allele frequency (MAF) > 5% in each analyzed population were used for iHS and XP-EHH analyses, respectively. Both statistics were normalized internally by the program as a function of their derived allele frequency. F_ST_ values were calculated from the phased, masked data using Plink (92) for each pair of populations (Tsimane versus Han Chinese, Tsimane versus Peruvians, Han Chinese versus Peruvians), and converted to a PBS score using the equation provided in ref. 5. Only variants with MAF > 1% in each of the three analyzed populations were considered in downstream analyses. To avoid false positives induced by analyzing data in regions of the genome that are inaccessible to short read sequencing technology, we removed loci in the 1000 Genomes Phase 3 strict mask from our iHS, XP-EHH, and PBS result set.

To identify candidate regions that have putatively been under positive selection, we used two approaches derived from the literature to combine our iHS, XP-EHH, and PBS results and identify outlier regions (see *SI Appendix*, *Materials and Methods*). The set of 50-kb candidate/outlier regions identified by our two summary approaches were highly overlapping (*SI Appendix*, Table S2). The union set with overlapping regions collapsed is summarized in *SI Appendix*, Table S3.

### Overlapping Candidate Genes with Published GWAS Results.

We considered candidate genes for positive selection to be protein-coding genes that overlapped each candidate window; for anyone who wants to consider a broader distance between genes and variants (e.g., that may be involved in long range regulatory interactions), we also provide a list of genes whose transcription start or end sites are within 250 kb of each candidate window in *SI Appendix*, Table S5. To understand which traits our set of overlapping candidate genes had been linked to, we used https://www.targetvalidation.org/. First, we looked up each candidate gene in the database and noted which traits it had been associated with in previous GWAS (at *P* < 5 × 10^−8^). Next, for traits associated with more than six genes in our dataset, we used a hypergeometric test to ask whether the number of candidate genes in the Tsimane that were also associated with the focal trait was more than expected by chance. In cases where several GWAS have investigated the same trait, we used summary statistics from the study with the largest sample size (*SI Appendix*, Table S6). To perform these tests, we used the R function *phyper* after calculating 1) the number of genes associated with any 50-kb window in our dataset, 2) the total number of candidate genes, 3) the number of genes associated with the focal trait at *P* < 5 × 10^−8^, and 4) the overlap between #2 and #3. Results from these analyses are presented in Fig. 3 and *SI Appendix*, Table S6. We only considered tests that passed a 10% false discovery rate (FDR) to be significant.

### Testing for Polygenic Selection on Immune-Related Traits.

Given the polygenic nature of most traits (32), we also searched for evidence of polygenic selection on immune-related traits using the approach described in (65). To do so, we used two a priori defined sets of genomic regions that have previously been associated with immune-related traits: 1) SNPs associated with 22 quantitative immune traits across diverse ancestries in the UK Biobank (downloaded from https://pan.ukbb.broadinstitute.org/) and 2) genes that code for structures known to biochemically interact with 16 families of viruses (obtained from ref. 35).

To test for polygenic selection on a particular quantitative immune trait or viral response, we first obtained all 50-kb windows associated with a given trait and summarized their genomic properties. We split the genome into nonoverlapping 50-kb windows, and for each window calculated 1) the total number of SNPs, 2) the number of conserved SNP positions [i.e., those that fell within Genomic Evolutionary Rate Profiling, aka GERP, conserved elements (93)], 3) the average recombination rate (using the 1000 Genomes Phase 3 genetic map), and 4) the median FCS. We considered a window to be associated with a trait from the UK Biobank if the window contained a significant SNP (*P* < 5 × 10^−8^ from a meta-analysis across ancestries). We considered a window to be associated with the response to a particular virus if it contained any portion of the coding region of a virus-interacting gene or any portion of the 10-kb putative regulatory regions upstream and downstream of a virus-interacting gene.

After we obtained all 50-kb regions associated with a given trait (i.e., a quantitative immune trait or a viral response), we generated a null distribution using two permutation schemes. In the first scheme (which is presented in the main text), we randomly sampled *x* windows (*x* being the number of windows associated with the trait) among windows with a similar number of total SNPs, similar number of conserved SNPs, and similar recombination rate. To generate this null distribution, we only sampled from windows that were not associated with the focal trait, and we defined “similar” as within the same quartile of the total SNP, conserved SNP, or recombination rate distribution. We performed 10,000 samples for a given trait, and then computed a *P*-value for each trait by calculating the proportion of resampled windows for which the median FCS value was higher than that observed for the real trait-associated windows. All *P*-values were adjusted for multiple testing using a Benjamini–Hochberg FDR approach (94), and traits passing a 10% FDR were considered to be under polygenic selection (*SI Appendix*, Tables S11 and S12). We found a significant result for basophil counts (*SI Appendix*, Table S12), but ultimately excluded this from our final results set; we did so because the trait was associated with an order of magnitude fewer testable loci in our dataset relative to all other quantitative immune traits (~17-fold on average) and was thus an extreme outlier.

In the second permutation scheme, we attempted to account for the fact that the set of 50-kb windows that overlap virus interacting proteins will not be randomly distributed across the genome (i.e., many will be adjacent to one another). Therefore, for a given family of viruses, we 1) randomly sampled the same number of genes associated with the response to that virus in our real dataset, 2) obtained all 50-kb windows that overlapped the protein coding or regulatory region of genes in this sampled list, and 3) calculated the median FCS value across all 50-kb windows. As in the first permutation scheme, we only sampled from windows that were not associated with the virus in our real dataset. We performed 10,000 samples, and then computed a *P*-value for each virus by calculating the proportion of samples for which the median FCS value was higher than that observed for the real virus-associated windows. All *P*-values were then adjusted for multiple testing (94). We provide the results of these permutations in *SI Appendix*, Table S11, and note that they agree with the primary permutation scheme results presented in the main text.

### Demographic Models and Neutral Genetic Simulations.

To confirm that the results we obtained from selection scans could not be explained by neutral processes, we inferred demographic parameters for the Tsimane and simulated genetic data in the absence of selection. We used fastsimcoal2 (56) to construct demographic models identical to those presented in figure S11 of ref. 16. We set parameters as fixed in cases where there were previously published estimates that are commonly used in the literature [e.g., timing of the out of Africa bottleneck, populations sizes for the 1000 Genomes populations (16, 95)]. We set unknown parameters about the demographic history of the Tsimane as open, and used historically and archeologically informed search ranges for Native American populations informed by (16). *SI Appendix*, Table S4 provides a description of all fixed and open parameters.

easySFS (https://github.com/isaacovercast/easySFS) was used to infer the joint, folded site frequency spectrum for the Tsimane, Peruvians, and Han Chinese using our set of unrelated and unadmixed individuals. As input loci, we used SNPs filtered for: nonautosomal SNPs, SNPs that were not biallelic, SNPs that were not in the Hardy–Weinberg equilibrium (*P* < 10^−8^), SNPs that were not genotyped across >90% of individuals, genic regions (defined as within coding regions or within 100 kb of any transcription start or end site), CpG islands, GERP conserved elements (93), and the 1000 Genomes Phase 3 strict mask. This filtering was performed to ensure that only well genotyped, putatively neutral regions of the genome were used for demographic inference.

Model parameters were estimated by running the model 100 times with 100,000 iterations per run. The best likelihood run was chosen and used to simulate 500,000 sites across 22 chromosomes for a dataset identical in sample size and composition to the one used to calculate the joint site frequency spectrum. We then calculated the iHS, PBS, and XP-EHH statistics from each SNP in the simulated data (using the same filtering criteria and approach as described for the observed data) and combined the three statistics into an FCS for each SNP. We performed these simulation and analysis procedures 100 times and combined the FCS values derived from all runs to create the distribution plotted in *SI Appendix*, Fig. S3. Finally, we compared the median FCS for each of our 21 candidate regions with the total distribution of FCS values observed under neutrality.

### mRNA-seq Data Generation and eQTL Mapping at Candidate Regions.

Between July and November 2017, venous blood samples were collected in PAXgene tubes from Moseten (n = 88) and Tsimane individuals (n = 154) and used to generate mRNA-seq data using the Lexogen QuantSeq 3′ FWD mRNA-Seq Library Prep Kit and the Illumina NovaSeq S4 platform. We used standard pipelines for read trimming (96), mapping (97), and compiling gene counts (98). Because the data were derived from whole blood, we removed the *HBB*, *HBA1*, and *HBA2* genes from our dataset. We then filtered for expressed, protein-coding genes, normalized the data with the R package limma (99) and corrected for known technical effects. Details of mRNA-seq data generation and processing are provided in the *SI Appendix*, Materials and Methods.

For each of the candidate regions putatively under positive selection, we mapped eQTL using the processed mRNA-seq data and MEGA genotype data for Tsimane and Moseten individuals. For each population, we removed SNPs with MAF < 5%, SNPs not in Hardy-Weinberg equilibrium (i.e., those with *P* < 10^−8^), and SNPs in strong LD (using the *indep-pairwise* function in Plink (86) and the same parameters described previously). For each gene associated with a 50-kb region putatively under positive selection, we then tested for associations between genotype and gene expression for all SNPs within the 50-kb window. We were only able to test for eQTL for genes that are expressed in whole blood (*SI Appendix*, Fig. S6). Association testing was performed using GEMMA (100), and included age and sex as covariates as well as a relatedness matrix inferred by the program from the filtered, genome-wide genotype dataset. Of all possible pairwise comparisons in the mRNA-seq dataset, 0.41% were inferred to have a relatedness value > 0.125 (corresponding to second degree relatives). For each SNP-gene combination, we extracted the *P*-value associated with the genotype effect and corrected for multiple hypothesis testing (101). We considered a SNP to be significant in the Tsimane and Moseten datasets if it exhibited an FDR < 10% in either population and a nominal *P*-value of at least 0.05 in the other population (see *SI Appendix*, Table S8 for summary statistics for significant eQTL).

### Mapping Genotype–Phenotype Correlations at Candidate Regions.

For each of our candidate regions putatively under positive selection, we also tested for genotype–phenotype associations using immune and metabolic biomarker data previously collected by the THLHP (see *SI Appendix*, Table S7 for sample sizes). For each candidate 50-kb window and each trait, we used GEMMA (100) to test for an association between genotype and phenotype controlling for age, sex, the first five PCs of the genotype matrix, and a random genetic effect defined by a genetic relatedness matrix. We included both PCs and a random genetic effect because previous work has shown that these model components capture different aspects of genetic structure (85, 102). For these analyses, SNPs were again filtered for MAF < 5%, SNPs not in Hardy-Weinberg equilibrium (i.e., those with *P* < 10^−8^), and SNPs in strong LD (using the *indep-pairwise* function in Plink (86) and the same parameters described previously).

For all tests associated with a given trait, we extracted the p-value associated with the genotype effect and corrected for multiple hypothesis testing (101). We considered a genotype–phenotype association to be significant if it passed a 10% FDR. We note that this approach results in a lower nominal p-value cutoff than is commonly used for GWAS (i.e., *P* < 5 × 10^−8^). However, our analyses are intended to provide evolutionary insight rather than to identify genetic variants for immediate diagnostic or clinical use. All genotype–phenotype associations passing the 10% FDR threshold are presented in *SI Appendix*, Tables S9 and S10 provides the top tag SNP (i.e., the SNP with the lowest p-value), for a given region and trait combination. In cases where several SNPs in LD were tied for the lowest p-value, we report all of them in *SI Appendix*, Table S10, and we randomly chose one for visualization. Finally, for all of the top tag SNPs reported in the main text and used for visualization, we also reran our analyses after permuting the genotype labels 1000 times; we did so to confirm that our empirical null distributions were uniform as expected (see *SI Appendix*, Fig. S8).

## Code Availability.

Analysis code is available at https://github.com/AmandaJLea/Tsimane_selection.

## Supplementary Material

Appendix 01 (PDF)

Dataset S01 (XLSX)

Dataset S02 (XLSX)

Dataset S03 (XLSX)

Dataset S04 (XLSX)

Dataset S05 (XLSX)

Dataset S06 (XLSX)

Dataset S07 (XLSX)

Dataset S08 (XLSX)

Dataset S09 (XLSX)

Dataset S10 (XLSX)

Dataset S11 (XLSX)

Dataset S12 (XLSX)

## Data Availability

Individual-level data are stored in the Tsimane Health and Life History Project (THLHP) Data Repository, and are available through restricted access for ethical reasons. THLHP's highest priority is the safeguarding of human subjects and minimization of risk to study participants. The THLHP adheres to the “CARE Principles for Indigenous Data Governance” (Collective Benefit, Authority to Control, Responsibility, and Ethics), which assure that the Tsimane and Moseten 1) have sovereignty over how data are shared, 2) are the primary gatekeepers determining ethical use, 3) are actively engaged in the data generation, and 4) derive benefit from data generated and shared for use whenever possible. The THLHP is also committed to the “FAIR Guiding Principles for scientific data management and stewardship” (Findable, Accessible, Interoperable, Reusable). Requests for individual-level data should take the form of an application that details the exact uses of the data and the research questions to be addressed, procedures that will be employed for data security and individual privacy, potential benefits to the study communities, and procedures for assessing and minimizing stigmatizing interpretations of the research results (see the following webpage for links to the data sharing policy and data request forms: https://tsimane.anth.ucsb.edu/data.html). Requests for individual-level data will require institutional IRB approval (even if exempt) and will be reviewed by an Advisory Council composed of Tsimane community leaders, community members, Bolivian scientists, and the THLHP leadership. A similar structure exists for the Moseten data. The study authors and the THLHP leadership are committed to open science and are available to assist interested investigators in preparing data access requests.
