# Supplementary material for: Natural selection of immune and metabolic genes associated with health in two lowland Bolivian populations
Source: Proc Natl Acad Sci U S A. 2022 Dec 27;120(1):e2207544120. doi: 10.1073/pnas.2207544120 (PMC9910614; doi:10.1073/pnas.2207544120)
Supplement: Supplementary file 1 — Appendix 01 (PDF) [file pnas.2207544120.sapp.pdf]

Supplementary Information for Lea et al. "**Natural selection of immune and metabolic genes associated with health in two lowland Bolivian populations**"

Supplementary items in this document:

- Supplementary Materials and Methods
- Supplementary Figure 1. Global ancestry analysis with ADMIXTURE
- Supplementary Figure 2. Manhattan plot of Fisher's combined scores
- Supplementary Figure 3. Observed selection statistics versus the distributions produced from neutral simulations
- Supplementary Figure 4. QQ-plots of genotype-phenotype association testing results
- Supplementary Figure 5. Phenotypic effects of genetic variation under selection, stratified by population
- Supplementary Figure 6. Whole blood gene expression levels for candidate genes
- Supplementary Figure 7. Moseten dataset overview
- Supplementary Figure 8. Permutation results for select genotype-phenotype associations
- Supplementary References

Additional supplementary items:

- Dataset S1. Unadmixed Peruvian samples from 1000 Genomes used for selection analyses
- Dataset S2. Candidate regions with evidence for positive selection (not collapsed)
- Dataset S3. Candidate regions with evidence for positive selection (collapsed)
- Dataset S4. Parameters used for demographic modeling
- Dataset S5. Genes overlapping or near candidate regions
- Dataset S6. Enrichment of genes that overlap candidate regions for trait associations in published GWAS
- Dataset S7. Sample sizes and demographic overview for the phenotypic dataset
- Dataset S8. Significant results from linear mixed effects models testing for eQTL in candidate regions
- Dataset S9. Significant results from linear mixed effects models testing for genotype-phenotype associations in candidate regions
- Dataset S10. Tag SNPs in candidate regions associated with phenotypic traits
- Dataset S11. Results from polygenic selection analyses
- Dataset S12. Results from polygenic selection analyses

Supplementary Materials and Methods

Genotype data filtering and processing

We used Plink [1] to remove the following SNPs from our total dataset of n=1286: non-autosomal SNPs, SNPs that were not biallelic, SNPs that were not in Hardy-Weinberg equilibrium ($p<10^{-8}$), and SNPs that were not genotyped across >90% of individuals. This filtering left us with 1,651,754 SNPs. We also removed 5 samples with call rates <90%, as well as 15 individuals with genome-wide heterozygosity values that were >3 standard deviations above or below the mean value for the sample set (as in [2]). For samples that were run in duplicate, we retained the replicate with the higher call rate.

Using these SNP and sample sets, we next performed analyses to create a dataset of unrelated Tsimane individuals for evolutionary inference. First, we removed SNPs with MAF<5% and sites in linkage disequilibrium. Specifically, we used the indep-pairwise function in Plink [1] to scan windows of 50kb with a 20kb offset, and to randomly prune variants within each window so that no pair exceeded an R^2 threshold of 0.8. We then estimated pairwise relatedness for all samples using PC-Relate, which performs well in sample sets with population structure [3]. We followed the workflow provided here: https://bioconductor.org/packages/release/bioc/vignettes/GENESIS/inst/doc/pcair.html. Using the relatedness estimates from PC-Relate and functions provided by the program, we randomly pruned the dataset so that no individuals remaining in the dataset exhibited a kinship coefficient >0.125 (corresponding to third degree relatives). This left us with 203 Tsimane individuals.

Next, we used GenotypeHarmonizer [4] to combine this reduced dataset with the 1000 Genomes Phase 3 call set [5] (downloaded from ftp.1000genomes.ebi.ac.uk/vol1/ftp/release/20130502/). This harmonization is necessary when combining genotype data generated via different platforms and pipelines, such that they are stored using potentially different or unknown strands. We excluded A/T and C/G SNPs that could not be unambiguously merged between the two datasets using the “ambiguousSnpFilter” option. This process resulted in 693,720 biallelic SNPs, which were then phased using Shape-IT v2.r904 [6] and the 1000 Genomes Phase 3 call set as a reference panel as well as the 1000 Genomes Phase 3 genetic map. We note that ideally a population specific reference panel and genetic map would be used for phasing, but given that none exist, we followed the common practice of using the 1000 Genomes dataset for analyses of genetically uncharacterized populations [7,8].

Principal components and admixture analyses

We merged our filtered, phased data with: 1) the 1000 Genomes Phase 3 call subset for Peruvians (the least admixed Native American population), Han Chinese (who are often used as the best available ancestral reference for Amerindians [8,9]), Yorubans (to represent African ancestry), and British (to represent European ancestry) and 2) all South and Central American populations from the Simons Genome Diversity Project [10]. We used Plink [1] to filter for MAF>5% in the merged dataset, remove sites in strong linkage disequilibrium (again using a window size of 50kb, a 20kb offset, and an R^2 threshold of 0.8), and to perform PCA (Figure 1).

We used two approaches to detect European and West African admixture. First, we used the program ADMIXTURE [11] to estimate the proportion of the genome originating from K ancestral populations for each individual, with K being specified a priori. We performed these analyses using the merged dataset described above for PCA, but with Han Chinese samples removed (such that the setup was similar to [8]). We ran ADMIXTURE with K=3-7. Analyses using a given value of K were run five times with different random seeds. For each value of K, we retained results providing the lowest cross-validation (CV) error and we report these result in Figure S1. For ease of visualization, reference populations with >20 samples were randomly pruned to n=20 and results for K=4 (the lowest K value close to the minimum CV error) are presented in the main text (Figure 2).

Our second approach relied on local ancestry assignments from RFMix [12]. Here, we used 1) British individuals to represent European ancestry, 2) Yoruba individuals to represent West African ancestry, and 3) all South and Central American samples from SGDP grouped together to represent Native American ancestry. The SGDP samples contain minimal European admixture and are therefore arguably a better reference set than the South and Central American samples from 1000 Genomes. Using these reference populations, we then assigned chromosomal segments for each sample to its most likely ancestral source. We also included Peruvian samples from 1000 Genomes in this test set as a positive control, given that admixture has been analyzed in this population previously [13–15].

We observe essentially no evidence for African ancestry within the Tsimane and minimal evidence of European admixture (Figure 2 and Figure S1). Our estimates of European admixture from RFMix are highly correlated with our estimates from ADMIXTURE (R^2=0.8393, $p<10^{-16}$; Figure 2C). For all downstream selection analyses, we pruned our set of 203 unrelated individuals down to 196 individuals with >95% Native American ancestry inferred from RFmix. We also pruned the 1000 Genomes Peruvian dataset down to 26 individuals with >85% Native American ancestry (using higher thresholds for Peruvians resulted in too few samples for analysis). The set of admixture-filtered Peruvian individuals used for selection analyses are

presented in Table S1. For the Tsimane samples that passed our filters, we followed the methods of [8] and masked SNPs in 1) regions of low confidence ancestry assignments (<90%) and 2) regions that were inferred to be inherited from a non-Native American ancestor (i.e., regions that passed our confidence threshold, but where the most likely assigned genetic source was European or African).

Summarizing selection statistics to identify candidate regions

To identify candidate regions that have putatively been under positive selection, we used two approaches to summarize our iHS, XP-EHH, and PBS results set. First, we rank ordered the |iHS|, XP-EHH, and PBS distributions and defined outliers as those in the top 1% of each distribution. We then binned the genome into 50kb windows with 25kb offsets, and counted the number of outlier loci for each statistic that fell in a given window. Windows with outlier numbers in the top 1% of the genome-wide distribution for all three selection statistics were considered as candidates. Because each statistic has its own underlying assumptions and sensitivities, windows that are outliers for many different tests are expected to be enriched for true positives [2,8,16]. We required all three statistics to exhibit outliers in a given region in order to identify the most robust signals; in other words, we aimed to minimize the false positive rate even if it came at the cost of a higher false negative rate (as is common in the literature [2,8,16]). We chose to use 50kb windows because simulation studies have shown this window size provides good power to detect sweeps with selection intensities between 2Ns=100 and 1000 [17].

Our second approach to identify candidate windows used the same results set, but summarized in a different way. Specifically, we combined the genome-wide rank of the three statistics for each SNP that was analyzable by all methods with a Fisher's combined score (FCS) [18]. This score was equal to the sum, over the three statistics, of $-\log_{10}$(rank of the statistic/number of SNPs tested; Figure S2). Outlier regions were then defined as those with a median FCS score among the highest 0.1% of the genome. We used this second approach because it has been shown that combining different neutrality statistics into a single score may increase power [18]. The rationale being that neutrality statistics are expected to be more correlated for positively-selected variants relative to neutral variants [19]. The set of 50kb regions identified by our two summary approaches were highly overlapping (Table S2) and the union set with overlapping regions collapsed is summarized in Table S3.

mRNA-seq data generation and processing

Between July and November 2017, venous blood samples were collected in PAXgene tubes from Moseten (n=88) and Tsimane individuals (n=154). Samples were processed according to manufacturer's protocol (kept at ambient temperature for 2 hours, then transferred to a -20°C, and were stored at -80°C after they were exported to the US). In the US, blood samples were sent to the UCLA Social Genomics Core Laboratory where RNA was extracted, prepared into libraries (using the Lexogen QuantSeq 3′ FWD mRNA-Seq Library Prep Kit), and sequenced on an Illumina NovaSeq to a depth of 6.99 ± 1.47 (SD) million reads per sample. Samples were sequenced in two batches, with all Moseten samples and five Tsimane samples sequenced in one batch and all remaining Tsimane samples sequenced in a second batch. Because of the extreme population-batch confound, we did not attempt to combine Tsimane and Moseten samples and instead processed them as two separate datasets (after removing the five Tsimane samples sequenced with the Moseten samples). Below, we describe our processing procedures, which were performed for each dataset separately.

Post-sequencing, reads were trimmed to remove adapter contamination using cutadapt [20], mapped to hg38 using the splice aware aligner STAR [21], filtered to only retain uniquely mapped reads, and overlapped with Ensembl gene annotations using HTSeq [22]. We subsetted the transcriptome to focus on protein coding genes, and removed the *HBB*, *HBA1*, and *HBA2* genes from downstream analyses because these genes were clear outliers with very high counts. The large number of reads assigned to hemoglobin related genes is not surprising given that the data were derived from whole blood samples.

For each protein coding gene (excluding *HBB*, *HBA1*, and *HBA2*), we calculated the median counts per million (CPM) value across all individuals and filtered for genes with median CPM >3. This left us with 11,295 protein coding genes (after excluding genes that only passed filters in one population). Read count data were then normalized using the function *voomWithQualityWeights* in the R package limma [23]. Further, we removed known technical effects—namely the proportion of uniquely mapped reads, the average length of aligned reads, the 260/280 ratio, and the number of total mapped reads—from each dataset using linear models in limma [23].

Supplementary Figure 1. Global ancestry analysis with ADMIXTURE. A) Cross-validation error obtained from 25 runs of the program ADMIXTURE with different random seeds and 5 values of K. K=5 consistently produced the lowest cross-validation error. Admixture results for B) K=4, C) K=5, and D) K=6. Each bar represents an individual, and the height of the colored bar on the y-axis denotes the proportion of the genome assigned to a given ancestry component. The number of colors in panels B-D corresponds to the number of *a prori* defined ancestry components for a given ADMIXTURE run; colors are recycled across panels for visualization, they are not related across different ADMIXTURE runs. GBR=British, PEL=Peruvian, and YRI=Yoruba individuals from 1000 Genomes; SGDP=all South and Central American individuals from the Simons Genome Diversity Project. 1000 Genomes populations were randomly subsampled to n=20 for visualization.

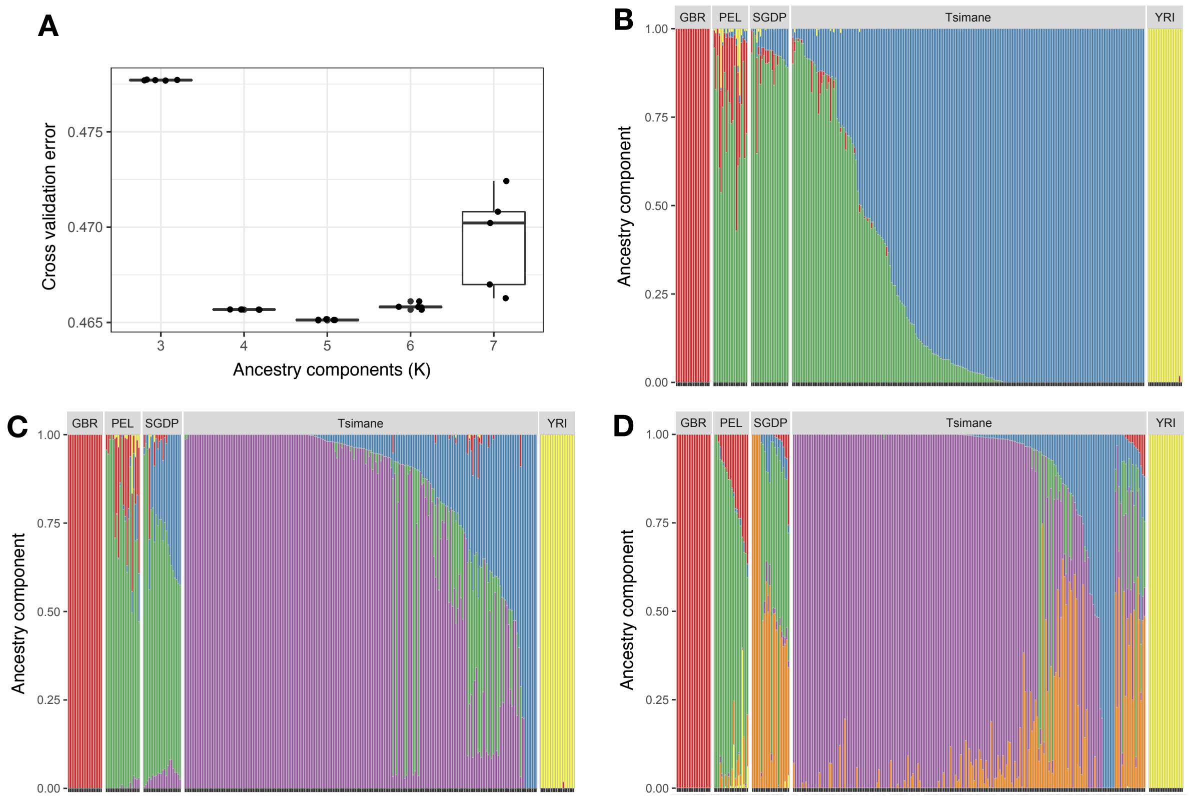

Supplementary Figure 2. **Manhattan plot of Fisher's combined scores**. Fisher's combined scores were calculated for 239,106 loci for which XP-EHH, iHS, and PBS could all be calculated.

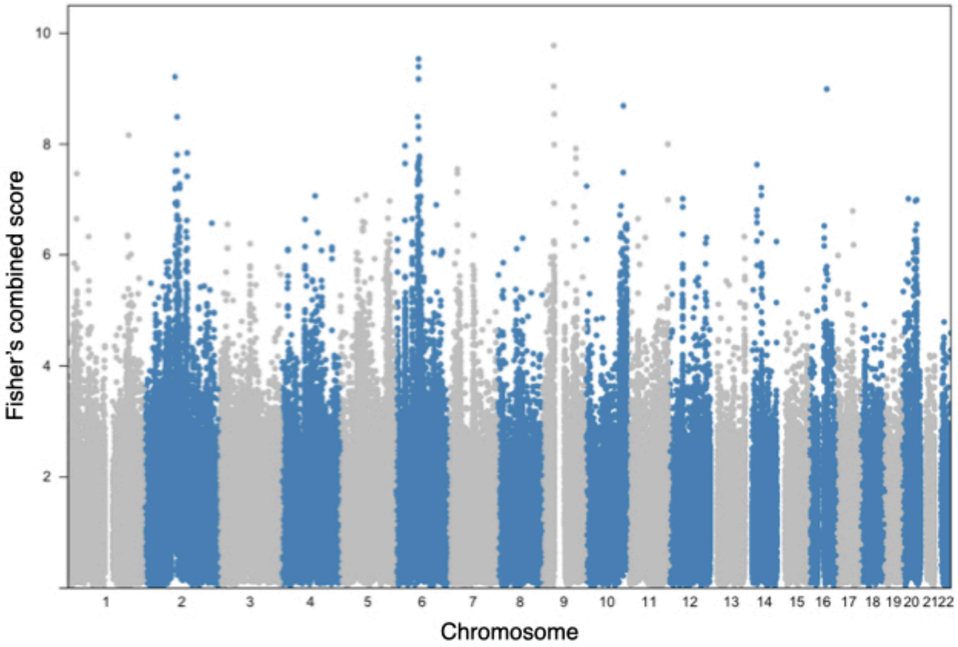

Supplementary Figure 3. Observed selection statistics versus the distributions produced from neutral simulations. The black line represents the genome-wide distribution of Fisher's combined scores obtained from 100 neutral demographic simulations. Fisher's combined scores (FCS) summarize the genome-wide rank of the iHS, PBS, and XP-EHH statistics for a given SNP (see Methods). The blue dots represent the median FCS for all SNPs in each of our 21 candidate regions. Points are jittered on the y-axis for visualization only (the y-axis values themselves are not meaningful).

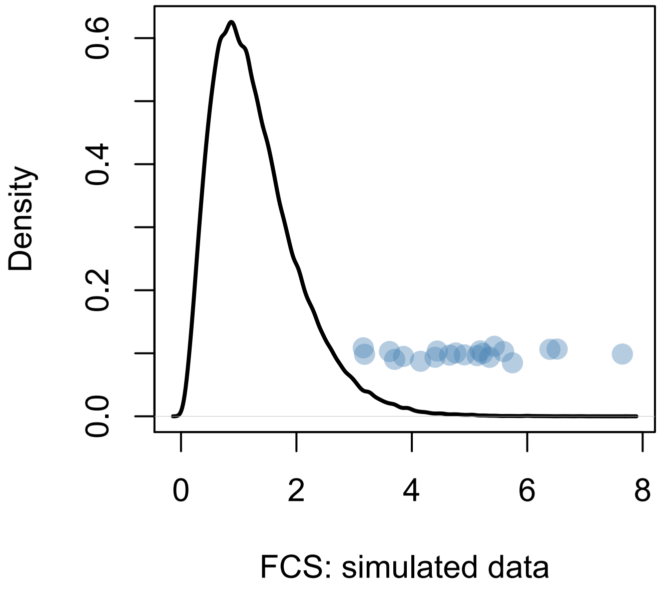

Supplementary Figure 4. QQ-plots of genotype-phenotype association testing results. Each set of colored dots compares the distribution of p-values obtained from performing genotype-phenotype associations in 19 candidate regions (y-axis) to the expected uniform distribution (x-axis). Dotted line represents x=y. A-B) Results from models that include both Tsimane and Moseten individuals, C-D) Results from models that include Tsimane individuals only.

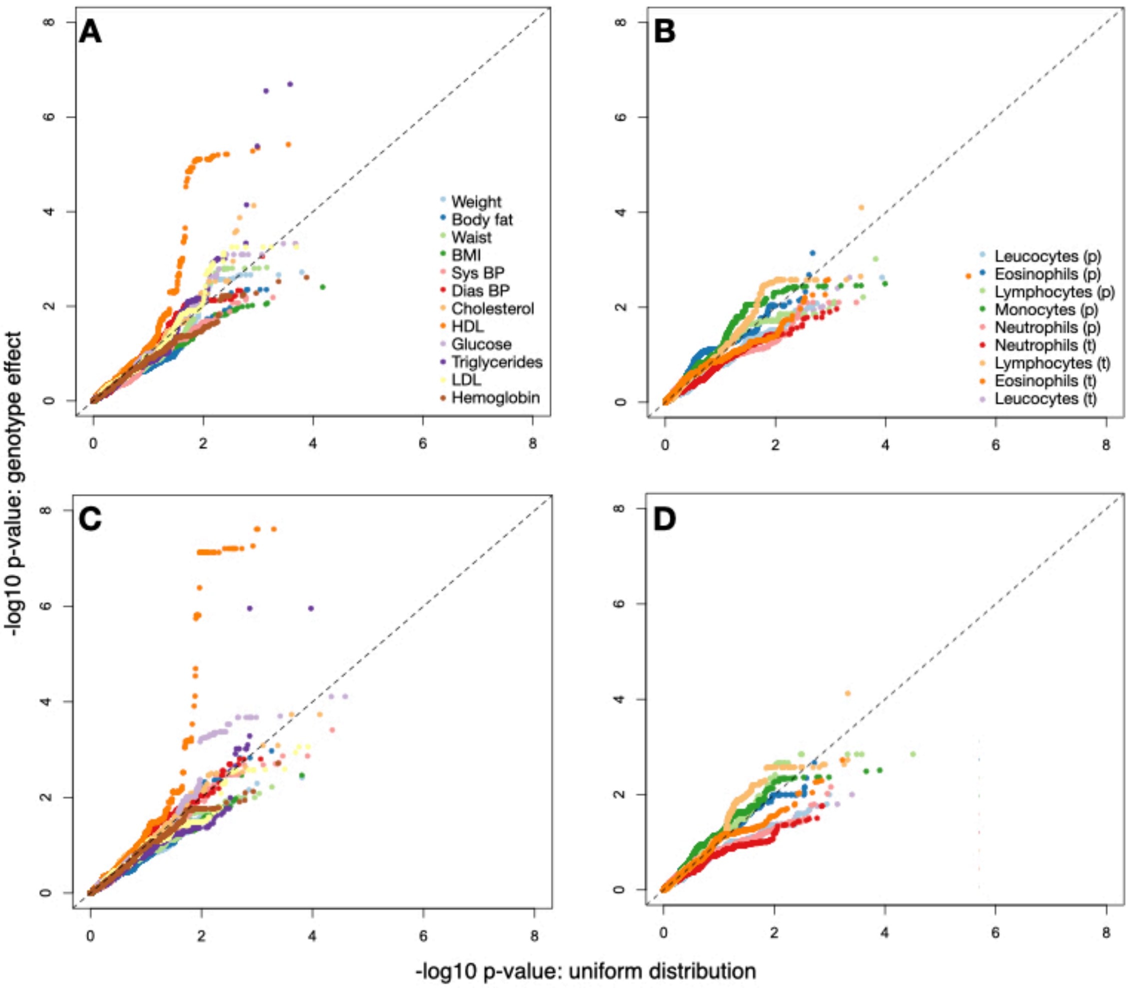

Supplementary Figure 5. Phenotypic effects of genetic variation under selection, stratified by population. A-B) Results for candidate regions with a significant eQTL, C-E) Results for candidate regions with a significant genotype-phenotype association. X-axis shows copies of the minor allele, y-axis shows normalized gene expression levels or phenotypic measures (units for triglyceride and HDL cholesterol levels=mg/dL). In all cases, gene expression and phenotypes were modeled linearly and assume an additive model. Detailed results are provided in Tables S9-10. SNPs used for association mapping are as in Figure 4.

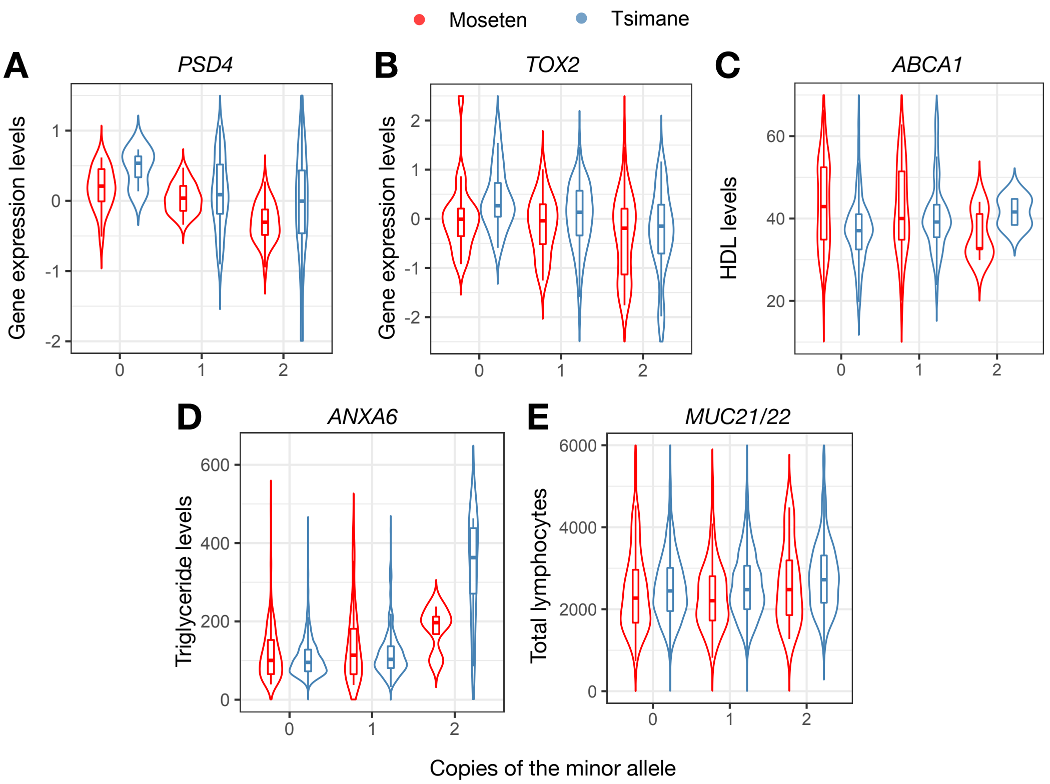

Supplementary Figure 6. Whole blood gene expression levels for candidate genes. X-axis shows all candidate genes and y-axis shows the distribution of log 10 counts per million (CPM) from RNA-seq data. A median CPM cutoff of >3 (equivalent to a log10 CPM>0.48) was used to filter for expressed genes.

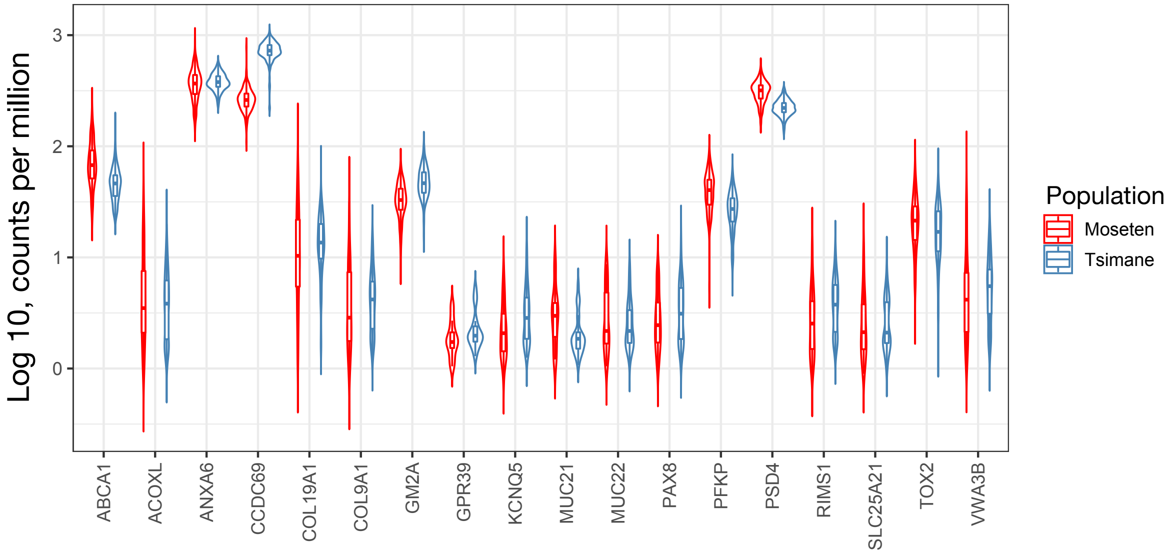

Supplementary Figure 7. Moseten dataset overview. A) Specific sampling locations within Bolivia for unrelated Tsimane (n=203) and Moseten (n=52) samples. B) Results from a principal components analysis including Tsimane and Moseten samples as well as 1) Han Chinese, Peruvians, Yoruba, and British individuals from 1000 Genomes and 2) all Central and South American individuals from the Simons Genome Diversity Project (SGDP). Samples are colored by their population of origin and shapes denote which study generated the data (circles=1000 Genomes, triangles=SGDP, squares=this study). Inset shows values for principal component 1 (PC 1) stratified by population and/or study for visualization.

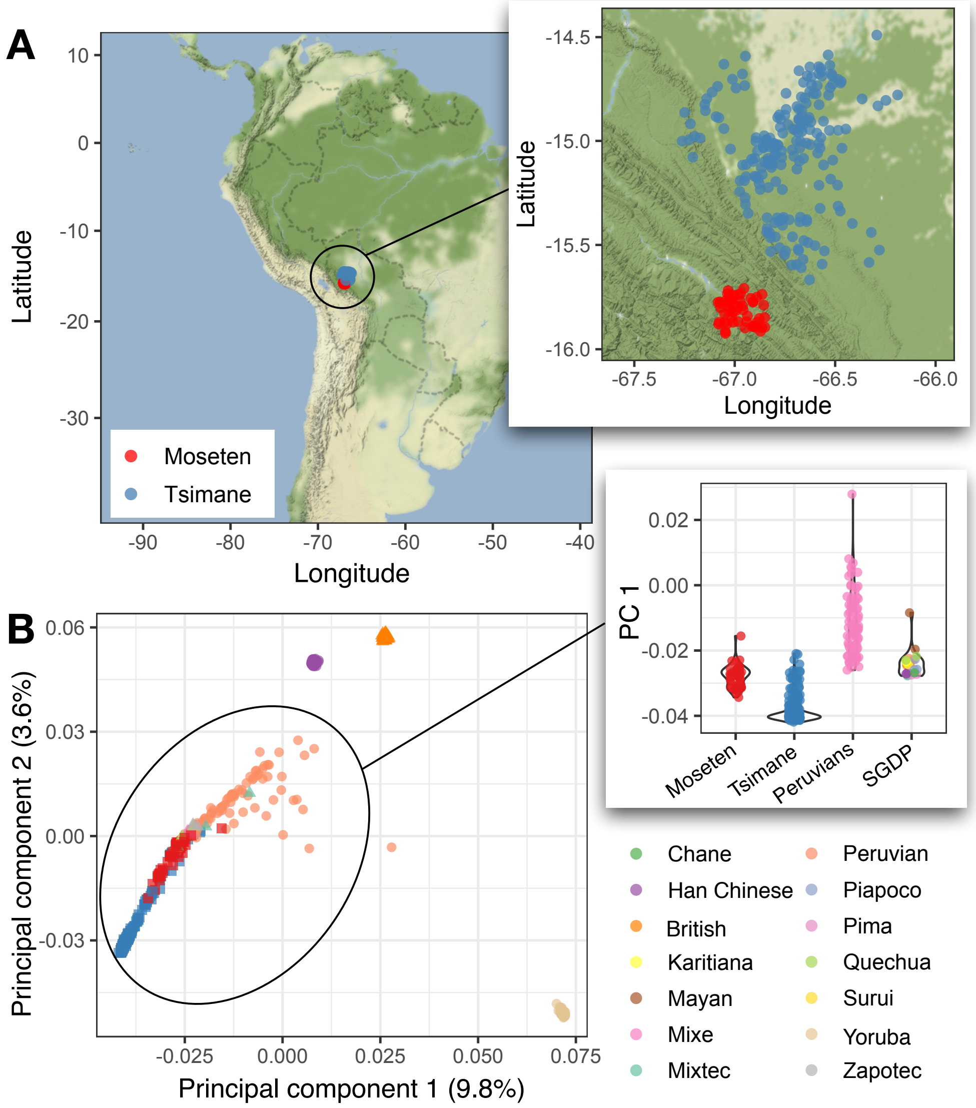

Supplementary Figure 8. Permutation results for select genotype-phenotype associations. For all tag SNPs discussed in the main text, we reran our analyses after permuting the genotype label 1000 times, to confirm that the empirical null distribution was uniform as expected. Histograms show the distribution of p-values for the genotype effect on a given phenotype from 1000 permutations.

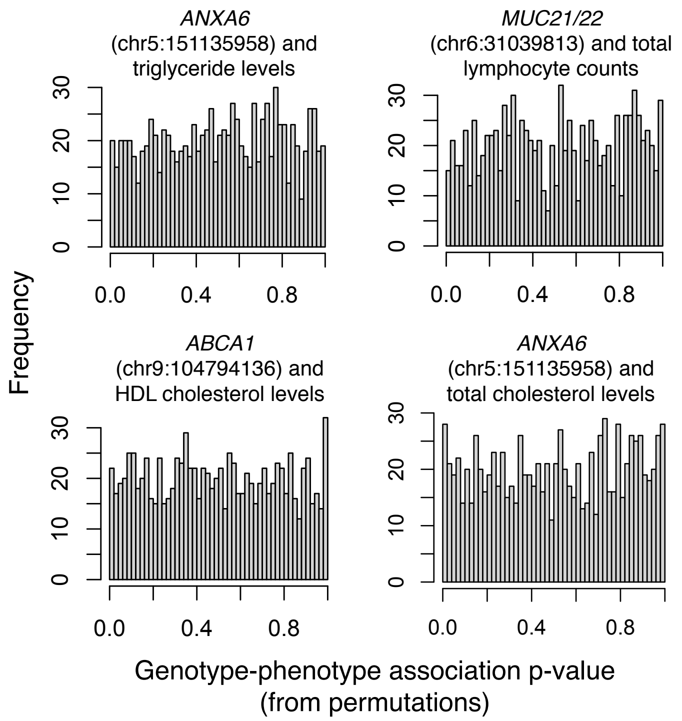

Supplementary References

1. Purcell S, Neale B, Todd-Brown K, Thomas L, Ferreira MAR, Bender D, Maller J, Sklar P, de Bakker PIW, Daly MJ, et al.: **PLINK: a tool set for whole-genome association and population-based linkage analyses**. *Am J Hum Genet* 2007, **81**:559–575.
2. Patin E, Lopez M, Grollemund R, Verdu P, Harmant C, Quach H, Laval G, Perry GH, Barreiro LB, Froment A, et al.: **Dispersals and genetic adaptation of Bantu-speaking populations in Africa and North America**. *Science (80-)* 2017, **356**:543–546.
3. Conomos MP, Reiner AP, Weir BS, Thornton TA: **Model-free Estimation of Recent Genetic Relatedness**. *Am J Hum Genet* 2016, **98**:127–148.
4. Deelen P, Bonder MJ, van der Velde KJ, Westra H-J, Winder E, Hendriksen D, Franke L, Swertz MA: **Genotype harmonizer: automatic strand alignment and format conversion for genotype data integration**. *BMC Res Notes* 2014, **7**:901.
5. The 1000 Genomes Project Consortium: **An integrated map of genetic variation from 1,092 human genomes**. *Nature* 2012, **135**:0–9.
6. Delaneau O, Coulonges C, Zagury J-F: **Shape-IT: new rapid and accurate algorithm for haplotype inference**. *BMC Bioinformatics* 2008, **9**:540.
7. Harrison GF, Sanz J, Boulais J, Mina MJ, Grenier J-C, Leng Y, Dumaine A, Yotova V, Bergey CM, Nsobya SL, et al.: **Natural selection contributed to immunological differences between hunter-gatherers and agriculturalists**. *Nat Ecol Evol* 2019, **3**:1253–1264.
8. Reynolds AW, Mata-Míguez J, Miró-Herrans A, Briggs-Cloud M, Sylestine A, Barajas-Olmos F, Garcia-Ortiz H, Rzhetskaya M, Orozco L, Raff JA, et al.: **Comparing signals of natural selection between three Indigenous North American populations**. *Proc Natl Acad Sci* 2019, **116**:9312–9317.
9. Lindo J, Huerta-Sánchez E, Nakagome S, Rasmussen M, Petzelt B, Mitchell J, Cybulski JS, Willerslev E, DeGiorgio M, Malhi RS: **A time transect of exomes from a Native American population before and after European contact**. *Nat Commun* 2016, **7**:13175.
10. Mallick S, Li H, Lipson M, Mathieson I, Gymrek M, Racimo F, Zhao M, Chennagiri N, Nordenfelt S, Tandon A, et al.: **The Simons Genome Diversity Project: 300 genomes from 142 diverse populations**. *Nature* 2016, **538**:201–206.
11. Alexander DH, Lange K: **Enhancements to the ADMIXTURE algorithm for individual ancestry estimation**. *BMC Bioinformatics* 2011, **12**:246.
12. Maples BK, Gravel S, Kenny EE, Bustamante CD: **RFMix: a discriminative modeling approach for rapid and robust local-ancestry inference**. *Am J Hum Genet* 2013, **93**:278–288.
13. Borda V, Alvim I, Mendes M, Silva-Carvalho C, Soares-Souza GB, Leal TP, Furlan V, Scliar MO, Zamudio R, Zolini C, et al.: **The genetic structure and adaptation of Andean highlanders and Amazonians are influenced by the interplay between geography and culture**. *Proc Natl Acad Sci* 2020, **117**:32557 LP – 32565.
14. Harris DN, Song W, Shetty AC, Levano KS, Cáceres O, Padilla C, Borda V, Tarazona D, Trujillo O, Sanchez C, et al.: **Evolutionary genomic dynamics of Peruvians before, during, and after the Inca Empire**. *Proc Natl Acad Sci* 2018, **115**:E6526 LP-E6535.
15. Price AL, Patterson N, Yu F, Cox DR, Waliszewska A, McDonald GJ, Tandon A, Schirmer C, Neubauer J, Bedoya G, et al.: **A genomewide admixture map for Latino populations.** *Am J Hum Genet* 2007, **80**:1024–1036.
16. Lopez M, Choin J, Sikora M, Siddle K, Harmant C, Costa HA, Silvert M, Mouguiama-Daouda P, Hombert J-M, Froment A, et al.: **Genomic Evidence for Local Adaptation of Hunter-Gatherers to the African Rainforest**. *Curr Biol* 2019, **29**:2926-2935.e4.
17. Enard D, Petrov DA: **Ancient RNA virus epidemics through the lens of recent**

adaptation in human genomes. *Philos Trans R Soc B Biol Sci* 2020, **375**:20190575.

18. Deschamps M, Laval G, Fagny M, Itan Y, Abel L, Casanova J-L, Patin E, Quintana-Murci L: **Genomic Signatures of Selective Pressures and Introgression from Archaic Hominins at Human Innate Immunity Genes**. *Am J Hum Genet* 2016, **98**:5–21.
19. Grossman SR, Shylakhter I, Karlsson EK, Byrne EH, Morales S, Frieden G, Hostetter E, Angelino E, Garber M, Zuk O, et al.: **A Composite of Multiple Signals Distinguishes Causal Variants in Regions of Positive Selection**. *Science (80-)* 2010, **327**:883–886.
20. Martin M: **Cutadapt removes adapter sequences from high-throughput sequencing reads**. *EMBnet.journal* 2011, **17**:10–12.
21. Dobin A, Davis CA, Schlesinger F, Drenkow J, Zaleski C, Jha S, Batut P, Chaisson M, Gingeras TR: **STAR: ultrafast universal RNA-seq aligner**. *Bioinformatics* 2013, doi:doi: 10.1093/bioinformatics/bts635.
22. Anders S: **HTSeq: Analysing high-throughput sequencing data with Python**. 2011,
23. Law CW, Chen Y, Shi W, Smyth GK: **Voom! Precision weights unlock linear model analysis tools for RNA-seq read counts.** *Genome Biol* 2014, **15**:R29.
